# Unexpected diversity of *Anopheles* species in Eastern Zambia: implications for evaluating vector behavior and interventions using molecular tools

**DOI:** 10.1038/srep17952

**Published:** 2015-12-09

**Authors:** Neil F. Lobo, Brandyce St. Laurent, Chadwick H. Sikaala, Busiku Hamainza, Javan Chanda, Dingani Chinula, Sindhu M. Krishnankutty, Jonathan D. Mueller, Nicholas A. Deason, Quynh T. Hoang, Heather L. Boldt, Julie Thumloup, Jennifer Stevenson, Aklilu Seyoum, Frank H. Collins

**Affiliations:** 1 Eck Institute for Global Health, University of Notre Dame, Notre Dame, IN, USA 46556; 2 National Malaria Control Centre, Chainama Hospital College Grounds, Off Great East road, P.O. Box 32509, Lusaka, Zambia; 3 Western Triangle Research Center, Montana State University, Conrad, MT, USA 59425; 4 Johns Hopkins Malaria Research Institute, Johns Hopkins Bloomberg School of Public Health, Baltimore, Maryland, USA.; and Macha Research Trust, Choma, Zambia; 5 Abt Associates, Africa Indoor Residual Spraying Project Ghana office, Accra, Ghana

## Abstract

The understanding of malaria vector species in association with their bionomic traits is vital for targeting malaria interventions and measuring effectiveness. Many entomological studies rely on morphological identification of mosquitoes, limiting recognition to visually distinct species/species groups. *Anopheles* species assignments based on ribosomal DNA ITS2 and mitochondrial DNA COI were compared to morphological identifications from Luangwa and Nyimba districts in Zambia. The comparison of morphological and molecular identifications determined that interpretations of species compositions, insecticide resistance assays, host preference studies, trap efficacy, and *Plasmodium* infections were incorrect when using morphological identification alone. Morphological identifications recognized eight *Anopheles* species while 18 distinct sequence groups or species were identified from molecular analyses. Of these 18, seven could not be identified through comparison to published sequences. Twelve of 18 molecularly identified species (including unidentifiable species and species not thought to be vectors) were found by PCR to carry *Plasmodium* sporozoites - compared to four of eight morphological species. Up to 15% of morphologically identified *Anopheles funestus* mosquitoes in insecticide resistance tests were found to be other species molecularly. The comprehension of primary and secondary malaria vectors and bionomic characteristics that impact malaria transmission and intervention effectiveness are fundamental in achieving malaria elimination.

With the call for malaria elimination, there is a renewed focus on understanding malaria transmission dynamics towards implementing more efficient and targeted intervention strategies^1^. At present, malaria interventions rely primarily on long-lasting insecticidal nets (LLINs) and indoor residual spraying (IRS). Both reduce malaria transmission by targeting mosquitoes that feed on human blood (anthropophagic), feed indoors (endophagic) and rest indoors (endophilic) and are therefore most effective in transmission systems where primary vectors feed indoors when people are asleep^1^,^2^. This was demonstrated in Kenya where primary vectors, *Anopheles funestus* and *An. gambiae*, which prefer feeding on humans and indoors, were shown to be more susceptible to LLINs and IRS, while the outdoor-biting *An. arabiensis* populations evaded intervention efforts^3^. Understanding small-scale vector bionomics is vital when malaria elimination is on the agenda^4^,^5^, as even small areas can act as strong foci for transmission.

Since vectors in many parts of the tropics may also be exophilic and exophagic, residual transmission may be sustained or extended by such secondary vectors - even when endophilic and endophagic vector populations are being controlled with IRS and/or LLINs^2^,^6^,^7^,^8^. Accurately identifying vectors and their bionomic characteristics are important when implementing effective interventions that also reduce residual transmission^1^,^9^.

The manner in which vector populations respond to these interventions and insecticide-associated selection pressures is not well understood and largely dependent upon the local vector composition. Control measures may be profoundly impacted by the development of physiological insecticide resistance^10^,^11^,^12^ and behavioral resistance - the ability of a vector population to change its bionomic characteristics in response to an intervention^13^,^14^,^15^,^16^,^17^. In the Solomon Islands, indoor residual spraying with dichlorodiphenyltrichloroethane (DDT) initially controlled all three primary vectors. Populations of the highly endophagic *An. punctulatus* and *An. koliensis* crashed and cannot be found today. *An. farauti* populations demonstrated a shift to earlier and outdoor biting thereby circumventing the indoor intervention strategy and maintaining a low level of residual malaria transmission^13^,^15^.

The two most prominent malaria vectors in Sub-Saharan Africa, *An. coluzzii* and *An. gambiae*, with varying insecticide resistance profiles, can only be distinguished by molecular techniques^18^. Adaptive introgression^19^ - where a malaria vector species may obtain an insecticide resistance gene via hybridization with a sister species, is particularly relevant to this study as sister-species population dynamics may determine the spread of genes conferring traits important to malaria transmission. Identifying other related, morphologically indistinguishable species in the same region could highlight such issues and allow downstream planning. Other known and understudied malaria vectors such as *An. rivulorum*^20^, *An. coustani*^21^,^22^,^23^, and their complexes require further bionomic characterization to be able to better understand their contributions to transmission.

Seventeen species of *Anopheles* (including several cryptic species) were identified using molecular techniques in the Kenya highlands^24^ - many of which were similar morphologically but displayed different bionomic traits. This demonstrates how unexpectedly complex transmission systems can be, even in areas that have been studied for many years. The presence of ‘unknown’ and novel vectors illustrates how the identification of some taxa can only occur when appropriate molecular techniques are used alongside detailed morphological identification. Epidemiology and entomology studies as well as vector control programs must be strategically based on an understanding of key local vector characteristics, such as feeding preferences and insecticide resistance, while also distinguishing vectors and non-vectors within anopheline cryptic species complexes, beyond the level of morphology^25^.

Malaria entomology studies in Africa usually focus on identifying members of two groups - the *An. gambiae* and *An. funestus* complexes, as historically these primary vectors have made up the majority of collections. This, however, often results in morphological misidentification or discarding of unexpected or novel vectors. Within well- and lesser- studied species complexes, new species are being discovered and phylogenies of these species are being further resolved using molecular techniques^18^,^22^,^26^,^27^,^28^. Collection sites may have diverse and unexpected anopheline populations, where detailed studies can expose diverse and unexpected arrays of secondary vectors^24^,^29^. Most studies rely on morphological identification with keys that may be outdated, contradictory, or difficult to interpret. This is further complicated by collections with damaged specimens, presence of new or cryptic species and species with overlapping characteristics, and intra-species morphological variation. Accurate morphological identification requires comprehensive training. By comparing molecular and morphological species identifications from different studies, we found that interpretations of species distributions, insecticide resistance assays, host preference studies, trap efficacy, and even screening for malaria parasites is largely skewed and sometimes entirely incorrect when using morphological identification alone. Identification of collections may bypass common mistakes and increase the level of taxonomic resolution, as well as contribute to a better association of species and their bionomics^24^.

Though this concept is not novel nor surprising for morphologically identical and similar sibling species, this analysis demonstrates misidentifications outside of species complexes. In this study, samples from several vector behavioral studies in high transmission areas of Luangwa and Nyimba Districts in eastern Zambia were analyzed using the ribosomal DNA internal transcribed spacer region 2 (rDNA ITS2) and the mitochondrial DNA cytochrome oxidase subunit 1 (mtDNA COI), quickly evolving and repeated regions, to molecularly differentiate and identify *Anopheles* species and members of species complexes^24^,^26^,^30^,^31^,^32^,^33^. This study represents the first in-depth look at species compositions in the Luangwa and Nyimba districts in Zambia. Identifying the primary and secondary malaria vectors in this area and their associated bionomic traits is vital for appropriate, targeted malaria control interventions and accurate monitoring of their effectiveness.

## Results and Discussion

### Molecular species determination

ITS2 sequences representing 1847 *Anopheles* mosquitoes were aligned into 18 sequence groups (hereafter called ‘species’) with a stringency of greater than 98% identity within each group to enable the maximum number of clusters. Small variations in ITS2 and COI sequences are strong indicators of distinct species. The majority of species-specific PCRs used to identify *Anopheles* and other cryptic species based on minor sequence variations within these regions^26^,^30^,^31^,^34^. ITS2 sequences from this study are available in GenBank with accession numbers KR014818 – KR014835.

Distinct sequence groups were arbitrarily called *Anopheles* species (AN) 1 through 18 prior to a more in-depth database comparison and species level identification. High similarity and the presence of voucher specimens allowed the identification of three species groups to species - AN12, AN14 and AN15 were *An. funestus* sensu stricto, *An. quadriannulatus,* and *An. arabiensis* respectively. Several species groups (AN3, AN4, AN5, AN10, AN11, AN13, AN16 and AN17) had high similarity to published sequences – but the absence of voucher specimens obviated an absolute species determination^24^. The presence of more than one species group that matched a particular species (E.g. AN5, AN6 and AN8 matched *An. coustani*; and both AN18 and AN4 were similar to *An. rivulorum*) indicated the presence of cryptic species or a complex of species. *An. coustani* s.s. has been shown to have a cryptic sibling species, *An. crypticus*^21^,^22^ - and our analysis indicates that, if one of these ‘*coustani*-like’ sequences belongs to *An. crypticus*, there may be an additional member in the complex. The sequences from the four groups (AN1, AN2, AN7 and AN9) did not share greater than 90% identity (when combined with sequence coverage) with any nr database sequence(s) (Table 1).

The COI sequences of 284 mosquito specimens were aligned and the resulting distinct contigs (18 species) were named as they correlated to the ITS2 groups (AN1-18). All COI sequence groups had a 1:1 relationship with the ITS2 groups, supporting the presence of 18 separate groups of anophelines. COI database searches identified AN4 as *An. rivulorum,* AN7 as *An. squamosus* and AN9 as *An. pharoensis.* When COI and ITS2 results were combined, AN2 was identified as *An. nili*, AN3 as *An. theileri*, AN5 as *An. coustani,* AN10 as *An. rufipes*, and AN13 as *An. longipalpis*. COI searches also identify several of the cryptic species seen as belonging within the *An. coustani* group (AN5, AN6 and AN8).

With limited database results, AN11, AN16 and AN17 were putatively described as *An. pretoriensis*, *An. maculipalpis* and *An. leesoni,* respectively. AN1 was not similar to anything in the databases but was identical to the sequences (both ITS2 and COI) of a currently unknown malaria infected *Anopheles* species in the western highlands of Kenya (“Species A”)^24^ (Table 1). These sequences are available in GenBank with accession numbers KR014836 – KN014853.

### Phylogeny

**Figure 1:**
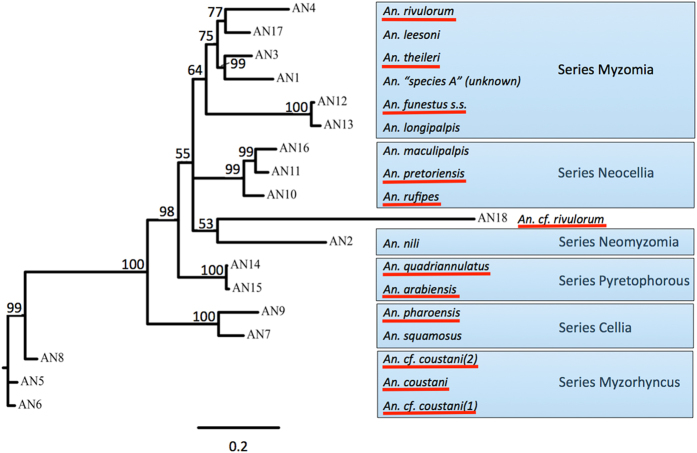
Phylogenetic Tree of ITS2 sequences. **A** majority rule consensus tree was generated from Bayesian analysis of dataset of ITS2 sequences. Bayesian posterior probabilities are shown above branches. AN18 is the only outlier and should cluster with Series Myzomia. Underlined species indicate those found positive with *Plasmodium spp.*

Consensus ITS2 sequences were aligned to construct a phylogenetic tree. The ITS2 tree (Fig. 1) groups putative species as expected based on their taxonomy. The *An. coustani* group (AN5, AN6 and AN8) cluster separately as part of the Myzorhyncus Series. *An. coustani* and members of this complex belong to subgenus *Anopheles*, whereas the rest of the species seen here are members of subgenus *Cellia*. AN14 and AN15 (*An. quadriannulatus* and *An. arabiensis*) cluster together as part of the Pyretophorus Series. The Neocellia series cluster is composed of AN10, AN11 and AN16 (*An. rufipes*, *An. pretoriensis* and *An. maculipalpis* respectively) while the Cellia Series cluster is composed of AN9 and AN7 (*An. squamosus* and *An. pharoensis*). The ‘funestus-like’ species (AN1, AN3, AN4, AN12, AN13, AN17) cluster as part of the Myzomyia Series while AN2 (*An. nili*) stands separate as part of the Neomyzomyia Series. The only exception is AN18 (*An. cf. rivulorum*) that does not group with AN4 (*An. rivulorum*) as expected. This might be due to the presence of several insertions and base-pair substitutions that would increase the divergence seen - though, without these changes, the sequence is highly similar to that of *An. rivulorum*. The COI sequences of these 2 groups are very alike and group together (data not shown).

Phylogenetic trees, combined with the stringent assembly and search procedures and the 1:1 match between ITS2 and COI sequences, all corroborate that these final species assignments (Table 1) are valid. Only members of cryptic species (AN6, AN8 and AN18) and those with no voucher specimens (AN1, AN11, AN16 and AN17) remain putative identifications. There are 18 species present in these collections, an unexpectedly high diversity of anophelines in a single region of eastern Zambia, particularly in light of most vector studies in East Africa focusing primarily on only known major mosquito vector species *An. funestus* and *An. gambiae* s.l, also present in this study.

### Molecular vs. morphological Identification

**Figure 2:**
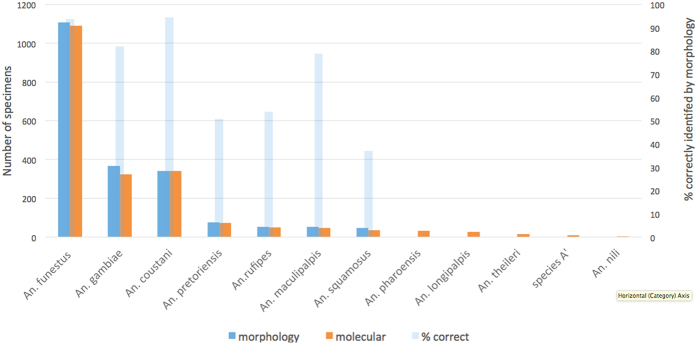
Comparison of morphological and molecular identifications. The number of specimens identified as specific *Anopheline* species by morphological and molecular techniques (n = 2024), and percentage accuracy of morphology compared to molecular identity are presented. Molecular identification was determined by sequencing of ITS2 and COI regions and comparisons to the database.

Species composition using morphological identifications was similar to that resulting from molecular analyses only for the most abundant species in the area, with *An. funestus sensu lato, An. gambiae* s.l., *and An. coustani s.l* comprising the majority of collections (53%, 16% and 17% of the sequenced specimens respectively) (Fig. 2). However, when studying individual specimens and less common species, a comparison of molecularly and morphologically derived species identities reveals that some samples were misidentified using morphological identification (Table 1). Most molecularly identified species were mistaken for multiple species in morphological identification. Morphologically, there were only 7 species identified, while sequencing demonstrated the presence of 18 species. Morphological identification did not identify any *An. theileri, An. pharoensis. An. nili,* or *An. longipalpis* specimens. Members of sibling species cannot be differentiated at this level. Of the three most numerous anopheline species groups caught, *An. funestus* s.l. (including *An. funestus* s.s., *An rivulorum*, *An. rivulorum-like,* and *An. leesoni*), *An. gambiae* s.l. (including *An. quadriannulatus* and *An. arabiensis)* and *An. coustani* s.l. (including *An. coustani* and two unidentified *An. coustani*-like species – *An. cf. coustani(1)* and *An. cf. coustani(2)*) 94%, 82%, and 94% respectively were identified correctly by morphology (Fig. 2). Correct identifications for the less common species ranged from 37% (*An. squamosus*) to 79% (*An. maculipalpis*). Assuming *An. funestus* s.l. and *An. gambiae* s.l. act as the major vectors in this area, 6% were missed by morphology and 10% of specimens were incorrectly assigned as these vectors. This does not take into account incorrect assigning of vector status for those cryptic species within species complexes that do not transmit malaria (see section below). In general, morphological identifications were more reliable for the more common species and those commonly assumed to be vectors or secondary vectors^24^.

### Impact on study analyses

The impact of morphologically indistinguishable specimens (cryptic species or members of a species complex) can be dramatic when trying to understand vector bionomics in a transmission system. The presence of specific *An. gambiae* s.l. complex members in Kenya and the differential effect interventions had on the three primary vectors (*An. gambiae* s.s.*, An. funestus* and *An. arabiensis*) explained both the reduction and persistence of malaria transmission in the presence of high intervention coverage^3^. Morphological identification alone could not have attributed persistence of transmission to a shift in species composition of the *An. gambiae* complex.

Contrasting behaviors seen within members of cryptic species^17^,^20^,^25^,^26^,^27^,^35^,^36^,^37^ and subpopulations within a described species may further complicate the understanding of intervention effectiveness. In the current study, the analysis of several bionomic traits was affected by the differences seen between morphological and molecular identification (Table 2).

#### Sporozoite PCRs

When determining species identity morphologically, only 4 species (*An. funestus* s.l., *An. gambiae* s.l., *An. coustani* and *An. pretoriensis*) were identified as carrying sporozoites while the molecular analysis identified 12 of the 18 molecular species positive for sporozoites (Table 1). A larger than expected number of sporozoite positive mosquitoes was found in secondary vectors, including *An. rivulorum* s.l. (2/30), *An. theileri* (2/14) and the *An. coustani* group (12/340).

Sporozoite PCR screening^38^ of 6,775 of 17,934 samples, excluding the insecticide resistance samples, enabled the detection of *Plasmodium* DNA in mosquito heads and thoraxes. A positive result does not necessarily indicate the species to be vector (i.e. infectious) but does point to this possibility, as analysis of the head and thorax alone would not pick up parasite DNA from infected midguts. A total of 222 of the 6,775 samples were positive for sporozoites (Table 1). This elevated rate of positives for each molecularly identified species is partly due to the non-random nature of the molecular sampling. A proportion (all or up to 96 per collection (period, village, study)) of each morphologically identified species underwent both molecular analysis as well as sporozoite analysis. The remaining mosquitoes all underwent sporozoite analysis with only samples with a positive sporozoite result being added to the molecular analysis. Accurate rates of parasite infection in the field, therefore, cannot be inferred from these data, though it is clear that more species than expected were positive for sporozoites.

Both the number of *An. funestus* s.s. collected (55% of specimens analyzed) and the high number of sporozoite infections seen (n = 185) confirms it to be the primary vector in this area^39^,^40^,^41^,^42^. Other known vectors, *An. arabiensis*^42^,^43^, the *An. coustani* group^6^,^7^,^23^, and *An. pharoensis*^6^,^7^,^44^ were also positive for sporozoites. Several species that are not considered primary vectors and some whose vector status is unknown were positive for sporozoites. These included *An. rivulorum* and its putative sibling species, *An. cf. rivulorum*, *An. rufipes*, *An. pretoriensis*, *An. quadriannulatus,* and *An. theileri*^24^,^45^. Given the presence of *Plasmodium* DNA, these species may possess the latent ability to be epidemiologically important malaria vectors in this site^20^,^23^,^24^,^45^,^46^,^47^. *Anopheles nili*, *An. leesoni*, *An. longipalpis,* and *An. squamosus*, all known vectors^6^,^7^,^23^,^27^,^45^,^48^,^49^, were not found to be positive for sporozoites – though for some species very few specimens were available for testing (e.g. *An. nili*, n=2). Though no sporozoites were found in samples of AN1 (*An. “Species A”*), this mosquito has been shown to be positive for sporozoites by both csELISA, PCR, and sequencing^24^ and has the potential to be a vector here as well. Presumed non-vector *An. maculipalpis* was not found with sporozoites. When taking into account the presence of positive sporozoite PCRs and historical data, 17 of 18 species are potential malaria vectors at this site, of which, only two (*An. quadriannulatus* and *An. maculipalpis*) were not considered vectors. Identifying sporozoite positive mosquitoes from the field is often the first step in incriminating vectors.

#### Host preference

When looking at host preference profiles, morphological identification demonstrated that *An. funestus* s.l. were trapped more frequently near goats and cows than indoors (22.9%) as this species is commonly believed to be highly anthropophagic. The preference for animals was initially attributed to zoophilic members of the *An. funestus* complex. When sibling species were identified molecularly, however, only 41.2% of *An. funestus* s.s. were found in human-baited traps with the majority caught from animal baited traps, confirming *An. funestus* s.s. to be a less anthropophilic population than commonly assumed.

Morphologically identified *An. gambiae* s.l. (sibling species cannot be differentiated morphologically) preferred to bite cows and goats (92%) rather than humans (8%) but the molecular analysis demonstrated that 100% of *An. arabiensis* were found in the human baited traps and none were found in the animal baited traps. Consistent with published literature, about 4.8% of zoophilic *An. quadriannulatus* were found in the human traps and the remaining 95.2% were found in goat and cow baited traps. The large number of *An. quadriannulatus* (13% of all collections) and the presence of positive sporozoite PCRs may indicate a role in transmission even with a low percentage seeking humans^46^,^47^,^50^.

A similar effect was seen with the *An. coustani* s.l. samples where molecular identification teased apart the relative host choices within the species seen while pure morphological identification cannot. When molecular identifications were used, *An. coustani* was far more anthropophilic than its ‘sibling’ species with 50% being found on humans while *An. cf. coustani(1)* was found on the animal baits 78% of the time and *An. cf. coustani(2)* did not seem to have a significant preference for any of the three baits used (human, cow and goat). Morphologically, *An. coustani* s.l. did not have a preference for any bait. The high numbers of these three species combined with the presence of sporozoites and their bionomic differences indicates varying and niche roles in transmission in this area^23^.

#### Vector presence, trapping and species diversity

Both morphological and molecular species identification point to *An. funestus* being the primary mosquito being caught, with the LTs catching a higher number of non-*An. funestus* species. The molecular data demonstrates that about 36% and 11% of the mosquitoes caught by the LTs and Ifakara Tent Traps (ITT) respectively were non-*An. funestus* while morphological identification indicated that this number was 33% and 4% respectively. Types of traps that take advantage of their species-specific behaviors can more efficiently sample specific mosquito species. For example, the anthropophilic and endophilic *An. funestus* s.s. was efficiently trapped in the indoor–simulating and human-baited ITT trap. Over these trapping periods, molecular data demonstrated that the LTs trapped twice the number of species (15 species in LTs versus seven in ITTs) and more of the primary vector species of these sites. Unlike the LT, the ITT did not trap any *An. coustani* s.l. (3 molecular species) and, the LT trapped 5x more *An. gambiae* s.l. This demonstrates that specific traps may be selected for and utilized for species-specific studies. The ITT would be useful at this site for studies on *An. funestus* s.s. (the major vector) while the LT would be more useful for investigations of other species or species diversity.

Molecular analysis of samples from the comparison of LTs and ITTs (n=903), demonstrated that another major mosquito species normally present at this site (*An. quadriannulatus*) was not identified in either the LT or the ITT. This may be due to *An. quadriannulatus* being zoophilic and both traps used here are human or human habitat based. In 2011, *An. funestus* s.s. comprised the majority (85%) of human landing catch (HLC) trapped mosquitoes in the indoor-outdoor study, while only one specimen of *An. cf. coustani(1)* was seen (0.01%) in the sample of the collections analyzed. However this was reversed in 2012, where most mosquitoes were the three molecular species of A*n. coustani* (75%) and *An. funestus* s.s. was only 10% part of the collections. This could have been a shift in species compositions after the implementation of IRS insecticides in 2012 that might have affected the indoor feeding population of *An. funestus* s.s. A further in-depth analysis using molecular data is required to understand these differences.

An analysis of samples that underwent both morphological as well as molecular analysis from the indoor–outdoor study also demonstrates species differences - with the caveat that morphology alone is not sufficient to distinguish some sibling species of mosquito complexes. In 2011, all 188 samples from HLCs, ITT and LT catches were morphologically identified as *An. funestus* s.l., however, sequencing indicates the presence of nine species, of which *An. funestus* s.s. predominates. Anopheline samples (n=17; 9%) misidentified include *An. coustani*, *An. arabiensis, An. nili, An. pretoriensis, An. quadriannulatus* and *An. theileri*. *An. longipalpis* and *An. rivulorum* are morphologically similar^27^ to *An. funestus* and were identified as such. Sequencing data also demonstrates that the *An. funestus* s.s. population, at this site, bites both indoors as well as outdoors and that there is a high diversity in species at this site including the presence several species with *Plasmodium,* that had not been demonstrated before.

#### Insecticide resistance

Molecular identifications of *An. funestus* mosquitoes used for insecticide resistance tests demonstrated that both *An. longipalpis* and *An. arabiensis* mosquitoes (both susceptible to lambdacyhalothrin at this site) were included in the insecticide resistance tests for the primary vector *An. funestus* (which demonstrates resistance to some insecticides). A random sample of mosquitoes from each day of insecticide resistance testing was sequenced. There were 3 *An. longipalpis* mosquitoes out of 19 (15%) sampled from a set of 30 total mosquitoes; 2 *An. longipalpis* mosquitoes out of 14 sampled (14%) from a set of 15 total mosquitoes and a single *An. quadriannulatus* mosquito was seen out of 7 (14%) mosquitoes from a set of 20. This could lead to a misinterpretation the amounts of insecticide resistance within the population. This observation is particularly important when performing meta-analyses. Since insecticide-resistance tests are often performed on a small subset of samples and the results of those tests can be misinterpreted if there is a mixed species composition, molecular typing of samples used in these tests would be cost-effective and important for their accurate interpretation.

The collections from light traps and ITTs over time have the potential to look at the species-specific effects of IRS to understand their contributions to transmission and hence, improve intervention strategies. Between 2011 and 2012, clusters in Luangwa and Nyimba districts received IRS (deltamethrin, lambda-cyalothrin and formulations of pirimiphosmethyl). IRS would be expected to reduce the densities of indoor-biting and -resting mosquitoes and could potentially drive changes in the behaviors of the various local species, depending on a species’ insecticide resistance status and inherent behavior (particularly exo-endophily). Using molecular techniques to accurately attribute insecticide resistance mechanisms and bionomic traits to the correct species is essential for the understanding of these processes and determining the effectiveness of control strategies. Data based on only morphological identifications can result in misleading conclusions and, therefore, impact upon strategies for selection of vector interventions and choice of insecticides.

It is important to note that the three major mosquito populations identified morphologically here - *An. funestus* s.l.*, An. gambiae* s.l. and *An. coustani* s.l. – are all species complexes that consist of vectors with unique sets of bionomic traits that differentially contribute to transmission. The resolution of species identification offered by molecular techniques, even using only a subset of the collected samples, is vastly greater than what is seen with only morphological methods.

Even under the best conditions, the limitations of morphological identification combined with misidentifications, and the resulting lack of species-level detail, inhibit efforts to associate species with bionomic characteristics and can dramatically influence analyses of these studies. Downstream associations of vector status, entomological inoculation rates, and impacts on control based solely on morphologically identified mosquitoes may be very different^24^ from those obtained from more precise molecular identifications. Correct identification is particularly relevant when considering insecticide resistance and vector population level bionomic vulnerabilities to interventions. This study reinforces the importance of combining molecular identifications with morphological identifications and also points to gaps that lie between site-specific infrastructure and resources for molecular processing - morphological identification may be the only possible route at many research sites. Collaborations and building local infrastructure and capacity may help alleviate this disparity.

A site -specific preliminary molecular characterization of mosquito populations, using sequencing or available PCR diagnostics, may be used to complement morphological approaches to determine the most efficient and cost effective strategy for the required detail of vector identification. Mosquito species that can be well characterized morphologically without many misidentifications might not require further analysis while others (depending on vector status, contributions to transmission, study goals, etc.) may require further molecular analysis for confirmation of species identities.

This study points to the effects of inaccurate interpretation of species composition in vector behavioral studies and the importance of molecular species identification of vectors when characterizing and evaluating transmission systems. The thorough comprehension of vectors and their bionomic characteristics that impact both malaria transmission and intervention effectiveness are fundamental to the malaria elimination agenda.

## Methods

### Mosquito collections

Mosquitoes were collected in 2011 and 2012 in Luangwa and Nyimba districts, Zambia. Several studies and methods contributed to this collection of anophelines (n = 18,100). These included the use of human landing catches (HLCs), CDC-Light traps (LT) and Ifakara Tent traps (ITT) (n = 13,960)^51^,^52^,^53^, the evaluation of host choice using a Latin Square design (n = 1181)^40^, animal (cow and goat) baited tents in a host preference study using a cross-over design (n = 1661), and an indoor-outdoor behavior study (n = 1132). All mosquitoes were processed for morphological identification using appropriate keys^49^,^54^. *An. funestus* mosquitoes that were used for insecticide resistance testing (2010–11) (n = 166) in the same area were also included in the analysis.

### Sporozoite detection

All mosquitoes received at the University of Notre Dame (n = 5433; LT and ITT collections: n = 3370; host choice study: n = 968; host preference study n = 1342; indoor-outdoor behavior study: n = 1095; and insecticide resistance collections: n = 166) were analyzed for sporozoites. Dissected heads and thoraces of female anophelines were analyzed with a nested PCR assay to confirm the presence of *Plasmodium falciparum* and *P. vivax* DNA^38^.

### Molecular processing

A subset morphologically identified mosquitoes (n = 2040) from the collections above (Table 1) were sequenced at ITS2 and/or COI loci for species identification. DNA was extracted from individual specimens using a CTAB technique. Briefly, samples were individually homogenized in CTAB extraction buffer, DNA extracted with phenol and isopropanol and precipitated with ethanol. The ribosomal DNA internal transcribed spacer region 2 (rDNA ITS2) region was isolated by PCR using primers developed for differentiating other *Anopheles* species complexes^32^. The ITS2 region was amplified from genomic DNA using the ITS2A and ITS2B primers^32^. The primer sequences were as follows: ITS2A 5′-TGTGAACTGCAGGACACAT-3′ and ITS2B 5′-TATGCTTAAATTCAGGGGGT-3′. The PCR mixture contained 2.5 μl of 10X buffer, 200 μM of each dNTP, 0.5 units of Taq DNA polymerase, 0.75 μl of 10 pmol/μl each of forward and reverse primers, and 2 μl of DNA template prepared as above. The thermocycling conditions were as follows: 94 °C for 5 minutes, 30 cycles of denaturation at 94 °C for 1 minute, annealing at 52 °C for 1 minute, and extension at 72 °C for 2 minutes, with a final extension at 72 °C for 5 minutes.

The mitochondrial DNA cytochrome c oxidase subunit 1 (COI) gene was amplified using LCO and HCO primers^55^. The primers used were LCO 1490 (5′-GGTCAACAAATCATAAAGATATTGG-3′) and HCO 2198 (5′-TAAACTTCAGGGTGACCAAAAAATCA-3′). The 25 μl PCR mixture contained 2.5 μl of 10X buffer, 0.2 mM of each dNTP, 1.2 mM MgCl_2_, 0.5 units of Taq DNA polymerase, 0.75 μl of 10 pmol/μl each of forward and reverse primers, and 1 μl of DNA template prepared as above. The thermocycling conditions were as follows: 94 °C for 5 min, 5 cycles of denaturation at 94 °C for 40 s, annealing at 45 °C for 1 min, and extension at 72 °C for 1.5 min; then 30 cycles of denaturation at 94 °C for 40 s, annealing at 51 °C for 1 min, and extension at 72 °C for 1.5 min; with a final extension at 72 °C for 5 min.

All specimens were initially amplified with ITS2 primers. Those specimens that did not amplify with ITS2 primers, and specimens corresponding to every novel ITS2 sequence were amplified with COI primers. The amplified fragments were visualized by electrophoresis on a 1% agarose gel. The PCR product was purified using an enzyme cleanup: 2U of Exonuclease 1 (USB Corporation, Cleveland, OH), 1U of Shrimp Alkaline Phosphatase (USB), and 1.8 μl of ddH20 were added to 8 μl of PCR product. This mixture was incubated at 37 °C for 15 min, followed by 15 min at 80 °C to inactivate the enzymes. The PCR products were sequenced directly (with one of the PCR primers) using Sanger sequencing on ABI 3730xl DNA Analyzer platform (PE Applied Biosystems, Warrington, England).

### Sequence Analysis

Raw ITS2 sequences were initially aligned using the Seqman pro assembler (Lasergene v 10.1.1) with a minimum match of 95%. Assembled contigs were manually examined for insertions, deletions and repeat structures that may impact assembly, inflate divergence and decrease identity scores. These contigs were then further divided into sub-contigs based on consistent single nucleotide polymorphisms (SNPs). A limit of 98% identity was used to assemble ITS2 sequences into final “species groups”. Low quality or contaminated sequences were not included in the analysis. The consensus sequences of these ITS2 contigs were compared (BLASTn) to the NCBI nr database for species identification.

The COI sequences were similarly assembled and compared to the NCBI nr and BOLD^56^ databases for confirmation of species identities. Sequence groups (henceforth called ‘species’) were merged (minimum identity of 94%) when COI BLAST results indicated that they belonged to the same species for a final minimum match of greater than 95%. These sequences were compared (BLASTn) to the NCBI nr database.

### Species Identification

Sequences were analyzed without regard to morphological identification. Single sequence contigs were not included in this analysis. High sequence identity (99% or greater) to voucher specimen sequences in the database was primarily used for final species confirmation. COI and ITS2 database comparison results were combined to determine species when either COI or ITS2 alone did not produce significant results to voucher specimens^24^. An initial threshold of 98% was lowered to 94% for COI groups as mitochondrial lineages can diverge more than ITS2 sequences within a species^31^,^33^. COI group sequences with less than 98% identity that matched the same anopheline species reference sequence were collapsed into one group. Lower COI sequence similarity was observed within a final species group than ITS2 sequences. To validate the proper assembly of these sequences, manual inspections were performed to ensure that sequence differences such as insertions, deletions and repeat regions did not inflate divergence and decrease identity scores. The manually examined consensus sequences of each group were compared (BLASTn) to the NCBI nr database to identify species if possible. A high degree of similarity combined with the presence of voucher specimens allowed the identification of species groups to species.

### Phylogenetic analysis

ITS2 sequences were initially annotated in a web interface for ITS2 delimitation accessible at the ITS2-DB (http://its2.bioapps.biozentrum.uni-wuerzburg.de). This database utilizes comprehensive Hidden Markov Model (HMM) approach to define the boundaries (start and end positions) of the ITS2 region, by comparing to a conserved structural motif at 5.8S/28S rRNA regions. The ITS2 sequences were then aligned in MAFT^57^ using X-INS-i^58^. This alignment method detects conserved secondary structures in non-coding RNA sequences and is based on the Four-way Consistency objective function to build a multiple alignment by combining SCARNA algorithm for the initial pairwise alignments^58^. Sequences were aligned using default settings in OPAL^59^ as implemented in in Mesquite 3.01^60^. Separate analyses for each sequence was done using Bayesian approach in MrBayes v3.1.2^61^ using a general time reversible (GTR) substitution model (COI tree analysis is not shown). Each analysis was performed with two independent runs with four chains and each run was carried out for 10,000,000 generations with a sample frequency of 1000. The first 25% trees were discarded as burn-in and the posterior probabilities were estimated from the remaining trees to infer branch support.

## Additional Information

**How to cite this article**: Lobo, N. F. *et al.* Unexpected diversity of *Anopheles* species in Eastern Zambia: implications for evaluating vector behavior and interventions using molecular tools. *Sci. Rep.* **5**, 17952; doi: 10.1038/srep17952 (2015).

## Figures and Tables

**Table 1:** Overview of morphological identifications compared to molecular identifications.

Species Group	Morphology based species ID	Samples analyzed (ITS2, C01)	ITS2 sequence homology	CO1 sequence homology	Tentative Species ID	Final Species ID	Sporozoite PCR +ve (#)
AN1	a	8, 8	Unknown	Unknown	*An. “Species A”*	Unknown	No
AN2	a	2, 2	*An. nili*	*An. nili*	*An. nili*	*An. nili*	No
AN3	a,b	14, 7	*An. theileri*	Unknown	*An. theileri*	*An. theileri*	Yes (2)
AN4	a,b	30, 4	*An. rivulorum*	*An. rivulorum*	*An. rivulorum*	*An. rivulorum*	Yes (1)
AN5	a,c	56, 4	*An. coustani*	*An. coustani*	*An. coustani*	*An. coustani*	Yes (2)
AN6	a,b,c,f	194, 13	*An. cf. coustani (1)*	*An. coustani*	*An. cf. coustani (1)*	Unknown	Yes (9)
AN7	b,c,d,e,f,g	17, 28	Unknown	*An. squamosus*	*An. squamosus*	*An. squamosus*	No
AN8	b,c	89, 19	*An. cf. coustani (2)*	*An. coustani*	*An. cf. coustani (2)*	Unknown	Yes (1)
AN9	a,c,d,f,g,h,	31, 22	Unknown	*An. pharoensis*	*An. pharoensis*	*An. pharoensis*	Yes (2)
AN10	a,d,e,f	49, 27	*An. rufipes*	Unknown	*An. rufipes*	*An. rufipes*	Yes (1)
AN11	a,b,c,d,e,f,g	73, 23	*An. pretoriensis*	Unknown	*An. pretoriensis*	Unknown	Yes (1)
AN12	a,b,e	1040, 85	*An. funestus ss*	*An. funestus*	*An. funestus ss*	*An. funestus ss*	Yes (185)
AN13	a,b,c	26, 11	*An. longipalpis C*	*An. funestus*	*An. longipalpis C*	*An. longipalpis C*	No
AN14	a,b,c,d,f	246, 6	*An. quadriannulatus*	*An. quadriannulatus*	*An. quadriannulatus*	*An. quadriannulatus*	Yes (8)
AN15	a,b,d	77, 7	*An. arabiensis*	*An. arabiensis*	*An. arabiensis*	*An. arabiensis*	Yes (9)
AN16	a,b,c,d,f,g	48, 5	*An. maculipalpis*	Unknown	*An. maculipalpis*	Unknown	No
AN17	a,f	11, 11	*An. leesoni*	Unknown	*An. leesoni*	Unknown	No
AN18	a,b	2, 2	*An. cf. rivulorum*	Unknown	*An. cf. rivulorum*	Unknown	Yes (1)

Final species identifications are based on both ITS2 and COI comparisons. Morphologically based species IDs are as follows - a: *An. funestus* s.l., b: *An. gambiae* s.l., c: *An. coustani* s.l., d: *An. pretoriensis*, e: *An. rufipes*, f: *An. squamosus*, g: *An. maculipalpis* and h: *An. implexus*.

**Table 2:** Comparison of morphological and molecular identifications.

	Study/Collection				
	LT and ITT collections	Host choice Study	Host Preference study	Indoor-outdoor study	Insecticide resistance sampling
Morphological species identified (total in collection)					
*An. gambiae* s.l.	70 (5671)	178 (405)	69 (279)	40 (152)	—
*An. funestus* s.l.	692 (7583)	36 (53)	1 (960)	213 (704)	166 (393)
*An. coustani* s.l.	43 (231)	163 (697)	48 (262)	86 (233)	—
*An. pretoriensis*	52 (171)	1 (2)	22 (35)	—	—
*An. rufipes*	32 (139)	1 (1)	18 (31)	1 (1)	—
*An. squamosus*	14 (91)	11 (15)	16 (20)	5 (5)	—
*An. maculipalpis*	—	2 (4)	44 (100)	6 (6)	—
*An. implexus*	—	—	—	1 (1)	—
Molecular species identified					
*An. “Species A”*	8	—	—	—	—
*An. nili*	—	—	—	2	—
*An. theileri*	10	2	—	2	—
*An. rivulorum*	11	11	—	8	—
*An. coustani*	7	33	4	12	—
*An. cf. coustani (1)*	15	87	26	66	—
*An. squamosus*	13	9	10	3	—
*An. cf. coustani (2)*	9	39	30	12	—
*An. pharoensis*	24	—	—	7	—
*An. rufipes*	22	4	22	1	—
*An. pretoriensis*	42	—	30	1	—
*An. funestus* s.s.	638	17	34	190	160
*An. longipalpis C*	13	5	—	3	5
*An. quadriannulatus*	50	170	23	2	1
*An. arabiensis*	72	7	—	48	—
*An. maculipalpis*	—	7	39	2	—
*An. leesoni*	18	—	—	2	—
*An. cf. rivulorum*	1	1	—	—	—

This table compares morphological and molecular species identified in the 5 studies where *Anopheline* samples were obtained. The number of samples from each morphologically identified species that were molecularly examined from a particular study are followed by the total number of morphologically identified samples for that species (in parenthesis). The number of molecularly identified samples from each study is presented.
